# Supplementary material for: Nanostructures as indicator for deformation dynamics
Source: Nat Commun. 2025 Dec 8;16:10947. doi: 10.1038/s41467-025-67150-4 (PMC12686469; doi:10.1038/s41467-025-67150-4)
Supplement: Supplementary file 1 — supplementary information [file 41467_2025_67150_MOESM1_ESM.pdf]

Supplementary information for Nanostructures as indicator for deformation dynamics

Sarah Incel[1,*], Markus Ohl[2,‡], Frans Aben[3], Oliver Plümper[2,†], and Nicolas Brantut[1, 4]

[1] *GFZ Helmholtz Centre for Geosciences, Telegrafenberg, 14473 Potsdam, Germany*
[2] *Utrecht University, Princetonlaan 8A, 3584 CB, The Netherlands*
[3] *TNO, Princetonlaan 6, Utrecht, 3584 CB, The Netherlands*
[4] *University College London, Gower Street, London, WC1E 6BT, United Kingdom*
[‡] *now at Oxford Instruments GmbH, Borsigstraße 15a, 65205 Wiesbaden, Germany*
[†] *now at Faculty of Geosciences, University of Bremen, Bremen, Germany, and MARUM - Center for Marine Environmental Sciences, University of Bremen, Bremen, Germany*
[*] *Corresponding author: sarah.incel@gfz.de*

Mechanical data

During the onset of failure, sample WG12 undergoes dilatancy hardening, i.e., stabilisation of failure as the pore fluid pressure drops due to dilation (Figure S1). The resulting peak slip rate of the self-stabilised sample is 0.27 mm/s. Under the same effective pressure but a lower absolute pore fluid pressure, this stabilisation process ends once the pore fluid pressure drops to zero followed by fluid vaporisation and dynamic failure of the sample. As failure occurred faster than the sampling rate of 1 Hz, we estimate the peak slip rate to be $\gg$1.5 mm/s.

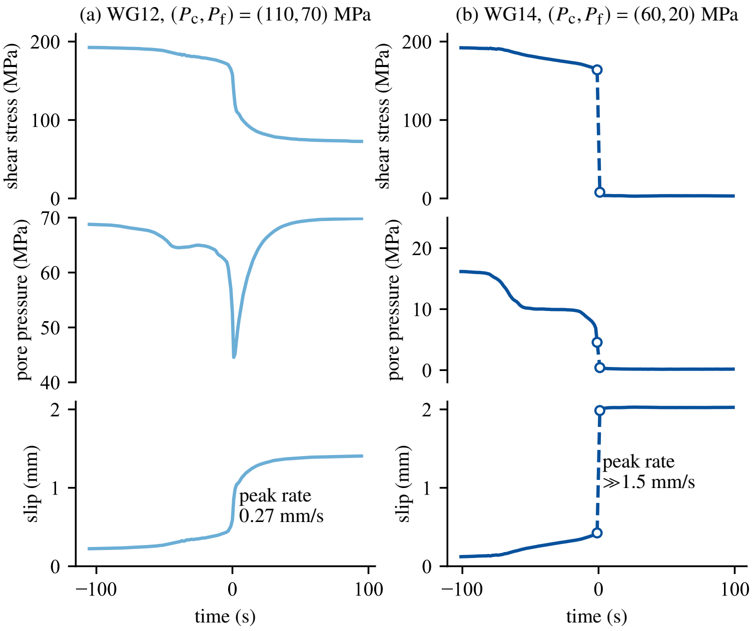

Fig. S1: Mechanical data for tests WG12 and WG14. Evolution of shear stress, pore pressure, and slip on the generated faults in samples WG12 (a) and WG14 (a), respectively.

Fault-gouge micro- and nanostructures

Here we exhibit further microstructures of the recovered deformation samples WG06 (controlled), WG12 (self-stabilised), WG14 (wet dynamic), and WG N03 (dry dynamic; Figure S2). Strikingly, we observe flow textures in all fault gouges as well as zones, in which individual grains can no longer be distinguished at the highest magnification at the scanning electron microscope (SEM; Figure S2). We therefore assume that these zones potentially contain amorphous material. These potentially amorphous zones are more frequent in samples that failed dynamically, i.e., wet dynamic (WG14) and

dry dynamic (WG N03), than in samples that failed slower in a controlled (WG06) or a self-stabilised manner (WG12). Furthermore, nanograins and vesicles, all associated to zones that are likely to be almost completely amorphous, are restricted to the two dynamic samples, i.e., WG14 (wet dynamic) and WG N03 (dry dynamic).

In addition, we provide nanostructures obtained by investigating a second focused-ion beam (FIB) foil cut from the wet dynamic sample (WG14; Figure S3). The location for this FIB foil is indicated in Figure S2c.

Analysis of spring-slider instability with inertia

In order to establish the potential stress overshoot due to piston inertia in our rupture experiments, we model the piston and rock sample as a spring-slider system, which has been shown to provide a good approximation of the slip dynamics in laboratory experiments (e.g. Johnson and Scholz, 1976; Shimamoto et al., 1980). We use a lumped-mass approximation whereby the piston and the upper half of the rock sample are considered to be a concentrated mass m in frictional contact with the static lower half of the rock supported by the base of the apparatus (Johnson and Scholz, 1976). We neglect damping effect, and do not consider wave propagation effects that can impact the dynamics of slip at the sub-millisecond timeframe (Lockner et al., 2017). The upper piston and rock sample is assumed to behave elastically, with a total stiffness k (in units of applied differential stress per unit shortening distance). The axial displacement u of the slider is given by Newton's law

$$m\frac{d^2u}{dt^2} = k(u_0 - u) - \sigma_\mathrm{f}, \tag{1}$$

where t is time, u_0 is the initial shortening at the onset of fault slip, and σ_f is the the strength of the fault (in terms of applied differential stress).

At the onset of slip, the fault is experiencing its peak strength σ_peak, so that static equilibrium imposes that $ku_0 = \sigma_\mathrm{peak}$.

With ongoing slip, our dry, quasi-static data show that the strength decreases down to a constant residual σ_res after a characteristic slip d_c, i.e., a shortening $u_\mathrm{c} = d_\mathrm{c}\cos\theta$ where θ is the fault angle with respect to the compression direction. We can describe the behaviour as a slip-weakening law,

$$\sigma_\mathrm{f} = \sigma_\mathrm{res} + f(u/u_\mathrm{c})(\sigma_\mathrm{peak} - \sigma_\mathrm{res}), \tag{2}$$

where $f(\cdot)$ is a nondimensional function that describes the shape of the slip-weakening curve. For simplicity, we choose here a linear decrease (see illustration in Figure S4a, solid line)

$$f(u/u_\mathrm{c}) = \begin{cases} 1 - u/u_\mathrm{c} \,\mathrm{if}\, u \leq u_\mathrm{c} \\ 0\,\mathrm{else}, \end{cases} \tag{3}$$

which is consistent with our experimental data in the dry, quasi-static test.

We can solve exactly the differential equation (1) with the strength evolution given by (2) and (3), and initial conditions $u(0) = u_0$ and an initial velocity $du/dt(0) = v_0$. The solution itself is of limited interest and will not be reproduced fully here. The main feature of the solution is that an initial steady acceleration phase occurs if $k < k_\mathrm{c}$, where $k_\mathrm{c} = (\sigma_\mathrm{peak} - \sigma_\mathrm{res})/u_\mathrm{c}$. This initial acceleration phase is followed by free oscillations of the slider. We are looking for the point of arrest of the slider, i.e., when slip rate becomes zero, and we want to extract the applied stress (in the spring) at this

point. In theory, this is the point where the slider would start sliding in the opposite direction, but in practice, we consider that backslip is not possible because it would require overcoming the frictional strength in the reverse direction.

The shortening rate in the first accelerating phase ($u < u_c$) is given by

$$\dot{u}_1(t) = v_0 \cosh(t/t_c \sqrt{1 - k/k_c}) \tag{4}$$

where $t_c = \sqrt{m/k_c}$. Shortening reaches $u = u_c$ at time

$$t_1 = \frac{1}{\sqrt{1 - k/k_c}} \text{asinh} \left(\sqrt{1 - k/k_c}/v_0 \right), \tag{5}$$

at which point the shortening rate is

$$\dot{u}_1(t_1)/v_c = \sqrt{1 - k/k_c + (v_0/v_c)^2}, \tag{6}$$

where $v_c = u_c/t_c$. In the second phase ($u > u_c$), the shortening rate is given by

$$\dot{u}_2(t)/v_c = \frac{1 - k/k_c}{\sqrt{k/k_c}} \sin \left((t - t_1) \sqrt{k/k_c} \right) + \sqrt{1 - k/k_c + (v_0/v_c)^2} \cos \left((t - t_1) \sqrt{k/k_c} \right). \tag{7}$$

The distance at which $\dot{u}_2$ becomes zero is found to be

$$u_{\text{final}}/u_c \approx \frac{1 + \sqrt{1 - k/k_c}}{k/k_c}, \tag{8}$$

where we neglected v_0/v_c^2 by comparison with k/k_c (which is valid when the initial shortening rate is negligible compared to the spontaneous, inertial oscillation rate of the slider).

The stress at $u = u_{\text{final}}$ is given by $\sigma_{\text{final}} = k(u_0 - u_{\text{final}})$, i.e.,

$$\sigma_{\text{final}} = \sigma_{\text{peak}} - (\sigma_{\text{peak}} - \sigma_{\text{res}})(1 + \sqrt{1 - k/k_c}), \tag{9}$$

so that the stress overshoot is

$$\Delta\sigma_{\text{over}} = \sigma_{\text{res}} - \sigma_{\text{final}} = (\sigma_{\text{peak}} - \sigma_{\text{res}})\sqrt{1 - k/k_c}. \tag{10}$$

The stress overshoot does not depend on the mass of the piston, but only on the stiffness of the loading system, the weakening rate of the fault and the strength drop. One can verify that the overshoot is zero for $k = k_c$, i.e., the load in the spring tracks exactly the strength drop, and that the overshoot is equal to the strength drop for highly compliant spring, or equivalently for instantaneous strength drop (Beeler, 2001). Figure S4a illustrates the strength and stress evolution for the case when $k = 0.5 \times (\sigma_{\text{peak}} - \sigma_{\text{res}}/u_c)$, where we see a substantial overshoot. The overshoot is greater than 80% of

the strength drop for stiffnesses smaller than around 0.36 of the weakening rate (Figure S4b). Limited overshoots of less than 20% of the strength drop only occur for spring stiffnesses within 0.96 of the weakening rate.

It should be noted that a more complete model of the triaxial apparatus would include damping effects, such as friction between piston and o-rings, or wave propagation effects (Lockner et al., 2017). Thus, our undamped model might predict an upper bound for the dynamic overshoot.

Nevertheless, using a simple model for the strength evolution observed under dry, quasi-static conditions, we predict that regular failure experiments (without any feedback to maintain quasi-static failure) should experience substantial overshoot and total stress drops, so that such total stress drops are not evidence for additional dynamic weakening processes.

References

Beeler, N.M., 2001. Stress drop with constant, scale independent seismic efficiency and overshoot. Geophysical Research Letters 28, 3353–3356.

Johnson, T.L., Scholz, C.H., 1976. Dynamic properties of stick-slip friction of rock. Journal of Geophysical Research 81, 881–888. doi:10.1029/jb081i005p00881.

Lockner, D.A., Kilgore, B.D., Beeler, N.M., Moore, D.E., 2017. The transition from frictional sliding to shear melting in laboratory stick-slip experiments, in: Thomas, M.Y., Mitchell, T.M., Bhat, H.S. (Eds.), Fault Zone Dynamic Processes: Evolution of Fault Properties During Seismic Rupture, Geophysical Monograph 227. first edit ed.. John Wiley & Sons, Inc., pp. 105–131.

Shimamoto, T., Handin, J., Logan, J.M., 1980. Specimen-apparatus interaction during stick-slip in a tri axial compression machine: A decoupled two-degree-of-freedom model. test Tectonophysics 67, 175–205. doi:10.1016/0040-1951(80)90234-6.

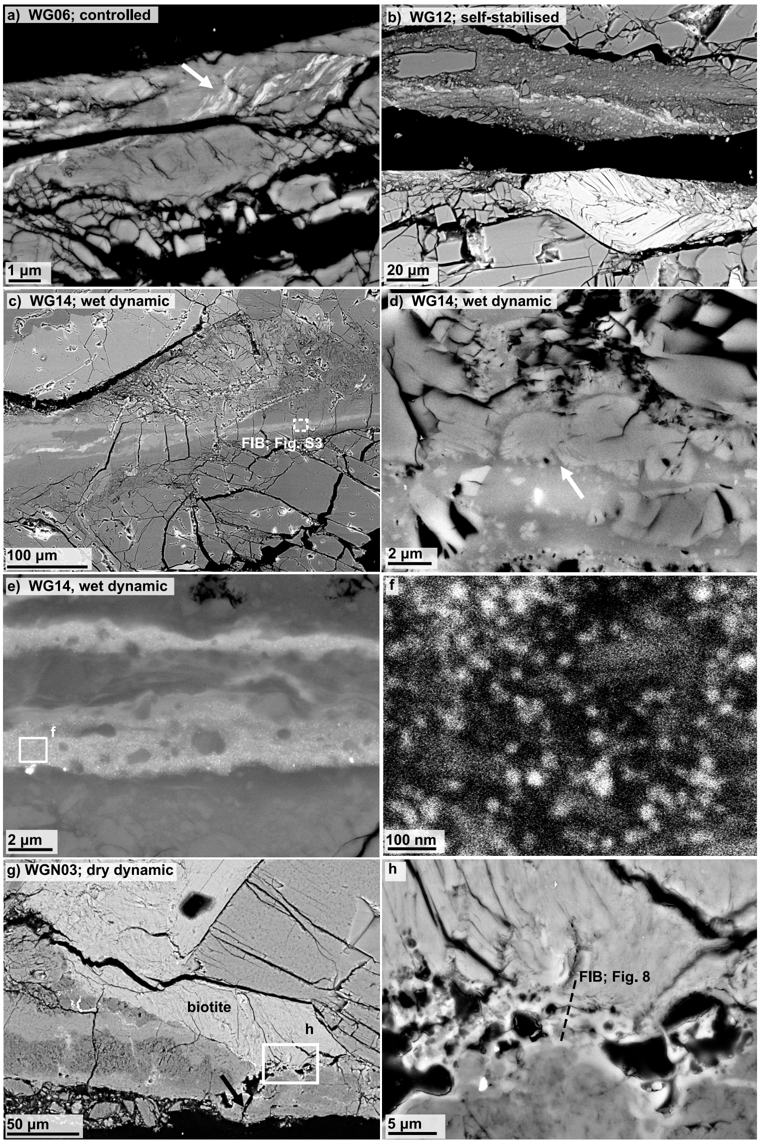

Fig. S2: Additional microstructural images of the deformed samples. All images were taken at the scanning electron microscope (SEM) in backscattered-electron (BSE) mode. The white arrow in image a) highlights flow structures created by sub-micron sized biotite flakes, which are visible at low magnification as well as exhibited in images b). White arrow in image d) points towards a feldspar fragment, which reveals sutured grain boundaries. High-magnification images of the zones marked in e) and g) show either numerous equisized nanograins (f) or versicles within amorphous-appearing material adjacent to a biotite grain (h).

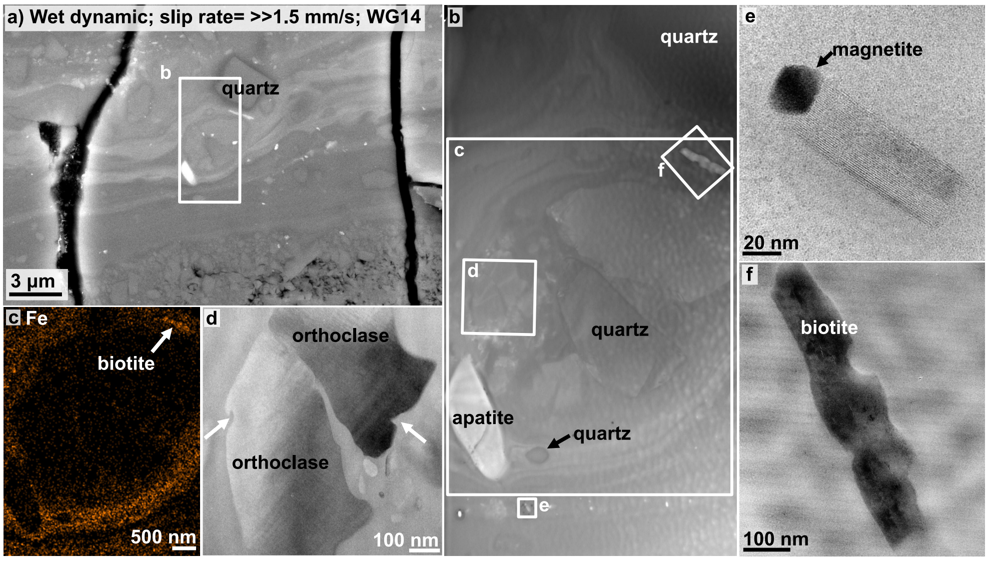

Fig. S3: Micro- and nanostructures of sample WG14. a) Backscattered-electron (BSE) image taken at the scanning electron microscope (SEM) showing the position of an additional focused-ion beam (FIB) section, which was a horizontal lift-out, marked by the white rectangle. b) Overview image taken in high-angle-annular dark-field (HAADF) at the transmission electron microscope (TEM) showing extensive flow structures of material appearing poorly crystalline, surrounding larger fragments identified as quartz, orthoclase, apatite, and biotite. c) The Fe element distribution map exhibits the variation in Fe content as a result of partial melting of biotite (f). d) Sutured grain boundaries of orthoclase grains showing their characteristic tweed structures. e) Euhedral magnetite grain adjacent to a biotite flake and surrounded by amorphous material.

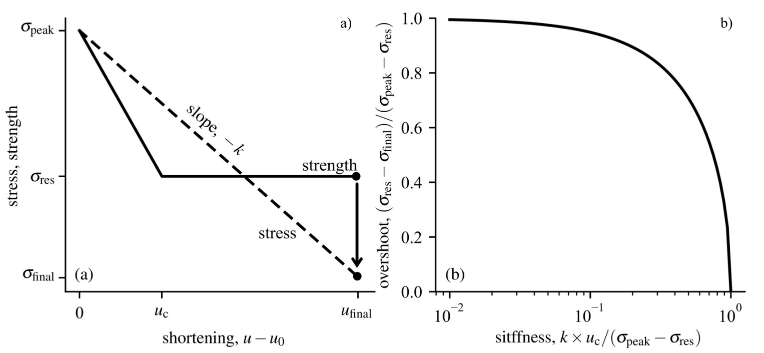

Fig. S4: (a) Stress (dashed line) and strength(solid line) as a function of shortening. For $k = 0.5 \times (\sigma_{\text{peak}} - \sigma_{\text{res}})/u_{\text{c}}$, slip arrests at u_{final}, after a significant stress overshoot. (b) Stress overshoot as a function of normalised stiffness. k= total stiffness; u= axial displacement; u_{c}= axial displacement after a characteristic slip d_{c}; σ_{peak}= peak stress; σ_{res}= residual stress; σ_{final}= final stress.
